# Effect of water source and feed regime on development and phenotypic quality in *Anopheles gambiae* (*s.l.*): prospects for improved mass-rearing techniques towards release programmes

**DOI:** 10.1186/s13071-019-3465-0

**Published:** 2019-05-06

**Authors:** Nwamaka O. Akpodiete, Abdoulaye Diabate, Frédéric Tripet

**Affiliations:** 1 Centre for Applied Entomology and Parasitology, School of Life Sciences, Keele University, Staffordshire, UK; 2 Institut de Recherche en Sciences de la Santé, Bobo-Dioulasso, Burkina Faso

**Keywords:** Water hardness, Feed, *Anopheles coluzzii*, *Anopheles gambiae*, Mass rearing, Insectary, Kisumu, Mopti, VK3

## Abstract

**Background:**

In many malaria-endemic sub-Saharan countries, insecticide resistance poses a threat to existing mosquito control measures, underscoring the need for complementary control methods such as sterile and/or genetically-modified mosquito release programmes. The sibling species *Anopheles gambiae* and *An. coluzzii* are responsible for malaria transmission in most of this region. In their natural habitat, these species generally breed in clean, soft water and it is believed that divergent preference in their larval breeding sites have played a role in their speciation process. Mosquito release programmes rely on the rearing of mosquitoes at high larval densities. Current rearing protocols often make use of deionised water regardless of the strain reared. They also depend on a delicate balance between the need for adequate feeding and the negative effect of toxic ammonia and food waste build-up on mosquito development, making managing and improving water quality in the insectary imperative.

**Methods:**

Here, we investigated the impact of water source and feed regimes on emergence rate and phenotypic quality of mosquitoes in the insectary. First-instar larvae of *An. gambiae* (Kisumu strain) and *An. coluzzii* (Mopti and VK3 strains) were reared in three water sources with varying degrees of hardness (deionised, mineral and a mix of the two), with a daily water change. Larvae were fed daily using two standardised feeding regimes, solution and powder feed.

**Results:**

Water source had a significant impact on mosquito size and development time for all strains. Earlier emergence of significantly larger mosquitoes was observed in mineral water with the smallest mosquitoes developing later from deionised water. Wing-length was significantly longer in mineral, mixed water and in powder feed, irrespective of sex, strains or water types. Deionised water was the least favourable for mosquito quality across all strains.

**Conclusions:**

Mineral water and powder feed should be used in rearing protocols to improve mosquito quality where the optimal quality of mosquitoes is desired. Although results obtained were not significant for improved mosquito numbers, the phenotypic quality of mosquitoes reared was significantly improved in mineral water and mix water. Further studies are recommended on the impact mineral water has on other fitness traits such as longevity, fecundity and mating competitiveness.

**Electronic supplementary material:**

The online version of this article (10.1186/s13071-019-3465-0) contains supplementary material, which is available to authorized users.

## Background

Despite the constant increase in malaria funding, this persistent, multifaceted disease was still responsible for 445,00 deaths in 2016, with 91% of these cases occurring in the African region [1]. Incidentally, that year, there was an increase of 5 million cases, from the 216 million cases reported in 2015 [1]. This illustrates how fragile are the gains made in our quest to control, and ultimately eradicate the disease. In the absence of effective vaccines and the evolution of resistance to available drugs by *Plasmodium* malaria parasites, vector control continues to be the most cost-effective line of defence as it interrupts the disease cycle by preventing the transfer of malaria-causing parasites to humans [2].

In sub-Saharan Africa, malaria is primarily transmitted by mosquitoes of the *Anopheles gambiae* complex with *Anopheles gambiae* and *Anopheles coluzzii* being the most abundant and widespread [3]. These morphologically indistinguishable sibling species co-occur over large areas of sub-Saharan Africa and do not exhibit intrinsic post-mating barriers to reproduction [4]. *Anopheles gambiae* (*s.s*.) is widespread throughout the region, extending across the continent all the way from West Africa, through Central and East Africa and into Madagascar. *Anopheles coluzzii* has a westerly distribution which spans from Northern Senegal (West Africa), West-central Africa and Angola (Southern Africa) [5]. In many of these regions, the two species are found in sympatry and are separated genetically by strong assortative mating, hence low hybridization rates [6, 7]. The first exception to this rule is some sympatric populations from far-West of Africa in Guinea-Bissau and The Gambia where hybrid frequencies as high as 22.9% have been recorded [8]. The second exception is populations in which the kdr-resistance allele of *An. gambiae* recently selectively introgressed into *An. coluzzii* which resulted in a temporary increase in hybrid-like genotypes [9, 10].

The current toolbox for controlling these prevalent vectors includes the use of insecticide-treated nets (ITN), indoor residual spraying (IRS) and integrated vector management (IVM) [1]. Although these methods have been very effective in the reduction of mortality and morbidity over the past decade, 50% of the countries with ongoing malaria transmission which were on track towards critical targets for reduction in mortality and morbidity, have recorded a stall in progress [1]. Insecticide resistance amongst other factors seems to stand out as a major driver for this change in trajectory. This current trend brings to the forefront the research for new vector control methods to complement the existing IVM techniques. The release of sterile or genetically-modified mosquitoes for the replacement suppression of mosquito populations is one such promising tool [11–13]. These approaches bear similarities with 1950s and 1960s sterile male releases, in that they involve the production of large number of males which by mating with wild females will cause either the decline in the target population over a short period [2] or its replacement with a population refractory to the malaria parasite [11]. Successful implementation of these techniques is based amongst other factors, on rearing protocols designed specifically for *An. gambiae* (*s.l.*), a species for which no large-scale release programme has ever been conducted. Since the species in this complex are particularly demanding in terms of water cleanness compared to culicid species [14, 15], the need for efficient water management whilst providing enough larval food for production is crucial. Equally important is the need for the resulting sterile or genetically-modified male mosquitoes to be of sufficient phenotypic quality to ensure optimal survival and mating competitiveness after release [2].

In nature, the sibling species, *An. gambiae* and *An. coluzzii* are thought to differ subtly in their preferred larval breeding sites [16]. Although larvae of both species can be found in the same habitat, *An. coluzzii* tends to prefer more permanent breeding sites often resulting from human activities such as irrigated rice fields, reservoirs, abandoned mines and quarries, deforestation, and drainage ditches [17]. In contrast, *An. gambiae*, whose populations usually peak during the rainy season, thrives in habitats that are more ephemeral and rain-dependent [5, 16, 18, 19]. Larval habitat divergence has repeatedly been cited as a possible driver of ecological speciation between these species and larval transplant experiments have shown that *An. coluzzii* avoids the aquatic predators associated with more permanent habitats more effectively [20, 21]. Whether *An. gambiae* develops better in the water with low mineral content such as rain filled pools is currently unknown. However, there is some evidence that *An. coluzzii* may tolerate water with a higher mineral content at least in some areas of Africa [22].

There is a dearth of information on the effect of water source (in relation to mineral content or hardness) on the development and phenotypic quality of *An. gambiae* (*s.l*.) in the laboratory. Deionised water is commonly used in mosquito rearing in the laboratory regardless of strain. Although, it has been demonstrated that different feed regimes affect mosquito phenotypic quality and development time [23, 24], these studies focused on the effect of feed in isolation and not in relation to different water sources. Some of these studies have linked food quality to development of larger mosquitoes which have higher fecundity and longevity especially in females [24, 25].

Here, we investigated the impact of different water sources (in relation to hardness) and feed regimes on the development and phenotypic quality of two well-established strains and one recently colonized strain of *An. gambiae* (*s.l*.) in the laboratory. First-instar larvae were reared in three water types with different levels of hardness and data were collected on larval survival, pupal survival and mortality, adult emergence, development time, wing-length and sex. Larvae were fed with food delivered as floating flakes, referred to as ‛powder feedʼ, or as liquid solution or ‛solution feedʼ. The results obtained show an improvement in mosquito phenotypic quality when mineral water is used, and this could be beneficial for mosquito rearing programmes small or large. These findings should lead to better protocols for mass-rearing of sterile or genetically-modified male anopheline mosquitoes towards releases for vector control.

## Methods

### Mosquito maintenance

All bioassays were conducted in dedicated insectaries of the Centre of Applied Entomology and Parasitology, Keele University, UK. The Kisumu strain of *An. gambiae*, colonized over 39 years ago, from the area of Kisumu, Kenya, East Africa; 14-year-old Mopti strain of *An. coluzzii*, colonized in 2003 by the Lanzaro Laboratory (UC Davis) from the village of NʼGabacoro droit near Bamako, Mali, West Africa, and a recently-colonised 1-year-old VK3 *An. coluzzii* strain from Vallee du Khou in Burkina Faso, West Africa (supplied by IRSS, Bobo Dioulasso), were used for the bioassays [26]. Mosquitoes were maintained at 25 ± 2 °C, relative humidity of 70 ± 5%, with a 12-h light/dark photocycle. Larvae were fed an optimized diet of ground fish food (Tetramin, Tetra, Melle, Germany) at a rearing density of 200 larvae/litre by manual counting [27]. Pupae were transferred to 5l plastic cages (*c.*20.5 cm height × 20 cm diameter), covered with netting for adult emergence. Cages had sleeved opening for easy management of mosquitoes and accessories. Approximately 600–800 adults were held in a cage, sugar was provided *via* a paper towel soaked in 10% glucose solution, and water *via* a soaked cotton pad in an upturned bowl placed on the cage netting. Female adult mosquitoes were fed with horse blood using an artificial feeding membrane (Hemotek feeding membrane system, Discovery workshops, Blackburn, UK). Styrofoam cups (egg cups) containing filter paper and water were placed in the cages four days post blood feeding, to collect eggs. Following the removal of the egg cups, the cages were washed thoroughly and sterilised with bleach. Mouth aspirators were used to transfer adults from one container to another when necessary.

### Experimental design

Ten first-instar larvae were placed in styrofoam cups containing 150 ml of water at 5 cm depth. Mosquitoes were reared in three water types with different levels of hardness: deionised water, mineralised water, and a 50:50 mix of both water types: (i) deionised water: sourced from the reverse osmosis unit installed in the laboratory; (ii) mineral water: bottled water containing minerals which are natural compounds formed through geological processes, sourced from a local shop with the following typical nutrient values/litre: calcium (11 mg), magnesium (3.5 mg), potassium (2.5 mg), sodium (10 mg), bicarbonate (25 mg), sulphate (11 mg), nitrate (15 mg), chloride (14 mg), dry residue at 180 °C (85 mg) and pH (6.2); and (iii) mix water: a 50:50 mix of deionised and mineral water.

Although water hardness is usually defined as the total concentration of calcium and magnesium in water in mg/l, it is caused by a variety of dissolved polyvalent metallic ions, mainly calcium and magnesium and other ions such as aluminium, barium, iron, manganese, strontium and zinc [28]. To determine the water hardness/nutrient content of the water treatments used, 42 readings of conductivity (µS), total dissolved solids (mg/l), and salinity (ppm) per treatment at 3 points during the experiment were taken using an EXTECH conductivity/TDS/salinity/Temperature hand-held meter (FLIR Commercial Systems, Inc., Nashua, USA). Mean values for TDS, salinity and conductivity of the three water types at 3 points during the study are shown in Table 1.

**Table 1:** Mean (95% CI) of total dissolved solids, salinity and conductivity: measure of water hardness

Water type	TDS (mg/l)	Salinity (ppm)	Conductivity (µS)
Deionised	27.55 (25.93–29.18) *42*	18.48 (17.38–19.58) *42*	39.54 (37.24–41.84) *42*
Mix	70.54 (68.97–72.12) *42*	47.47 (46.38–48.55) *42*	100.85 (98.58–103.12) *42*
Mineral	112.21 (110.53–113.89) *42*	75.78 (74.65–76.91) *42*	160.4 (157.99–162.80) *42*

*Note*: Sample size (*n* = 42) is italicized

Larvae were fed with two standardised feeding regimes (solution and powder feed). Powder feeding regime consisted of daily rations of ground fish food, using a spatula to spread on the water surface: 0.1 μl of Liquifry liquid fish food (Interpret Ltd, Surrey, UK) on day 1, 2 mg on days 2–3, 4 mg on day 4, and 10 mg on day 5 until pupation. Solution feeding regime consisted of the same food quantity dissolved in deionised water (0.1 μl of Liquifry on day 1, 0.1 ml of 1 g/50 ml of TetraMin Baby on days 2–3, 0.2 ml of 1 g/50 ml of TetraMin Baby on day 4, and 0.5 ml of 1 g/50 ml of TetraMin Baby on day 5 until pupation) and injected into the larval tray using a pipette.

The resulting balanced experimental design consisted of 3 strains × 3 water types × 2 feeding patterns × 10 replicates × 10 larvae per pot, for a total sample size of 1800 larvae. Larvae from each experimental group were transferred to fresh water (same water source as the original set-up) containers daily.

### Collection of data at life-cycle stages

Depending on the life-cycle stage of the mosquitoes the following data was observed and recorded: (i) larval survival: determined as the percentage of larvae that developed into pupae from the total number of larvae for each treatment; (ii) pupal survival: determined as the percentage of mosquitoes that emerged as adults from those that pupated in each treatment; (iii) pupal mortality: determined as the percentage of mosquitoes that died at the pupal stage from the total number of mosquitoes per treatment; (iv) adult emergence: determined as the percentage of mosquitoes that emerged as adults from the total number of larvae in each treatment; (v) development time: determined as the number of days from placement of first instar larvae in treatment cups until adult emergence; and (vi) wing-length: following emergence, adult mosquitoes were sexed and stored in 75% ethanol. One wing of all emerged adults was measured from the distal end of the allula to the apical margin (radius veins), excluding the fringe scale using a binocular microscope [29, 30]. A stage micrometer of 1 mm ruler length (Graticules Ltd, Kent, UK) was used for calibration on 2.5 magnification on a scale of 1 microscope unit = 0.04 mm).

### Statistical analysis

All data collected were analysed using the software JMP 13 (SAS Institute, Inc., Cary, North Carolina, USA). All data were checked for deviations from normality and heterogeneity, and analyses were conducted using parametric and non-parametric methods as appropriate. Replicate effects were tested and only reported when significant. Interactions between independent variables were tested using step-wise models and only those significant were retained in the final models. For analyses of proportion of larvae, pupae and adults, likelihood odds ratios were used for *post-hoc* pairwise group comparisons following logistic regressions. Body size was analysed through general linear models followed by Tukeyʼs HSD *post-hoc* pairwise comparisons. Finally, development times (day of emergence) were analysed by Cox Proportional-Hazard models and *post-hoc* Tukeyʼs HSD pairwise comparisons.

## Results

### Effect of water types and feed types on larval survival

**Fig. 1:**
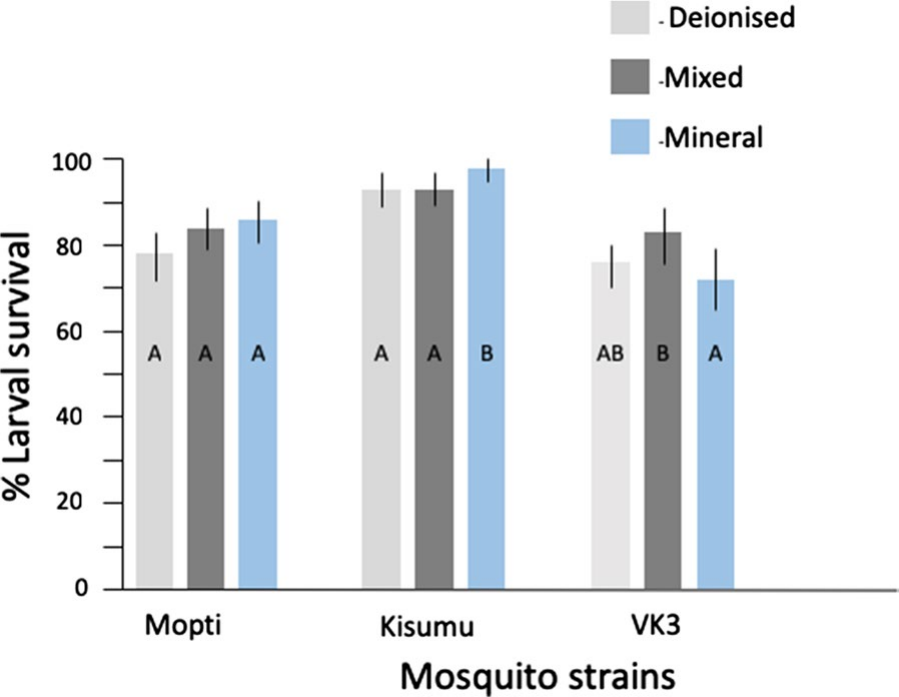
Effect of water source on larval survival. The percentage larval survival in deionised (light grey), mixed (dark grey) and mineral (blue) is shown across three strains of mosquitoes (Mopti, Kisumu, VK3). Whiskers represent 95% confidence intervals. Within strains, bar plots sharing a letter are not significantly different, those with different letters are significantly different

**Fig. 2:**
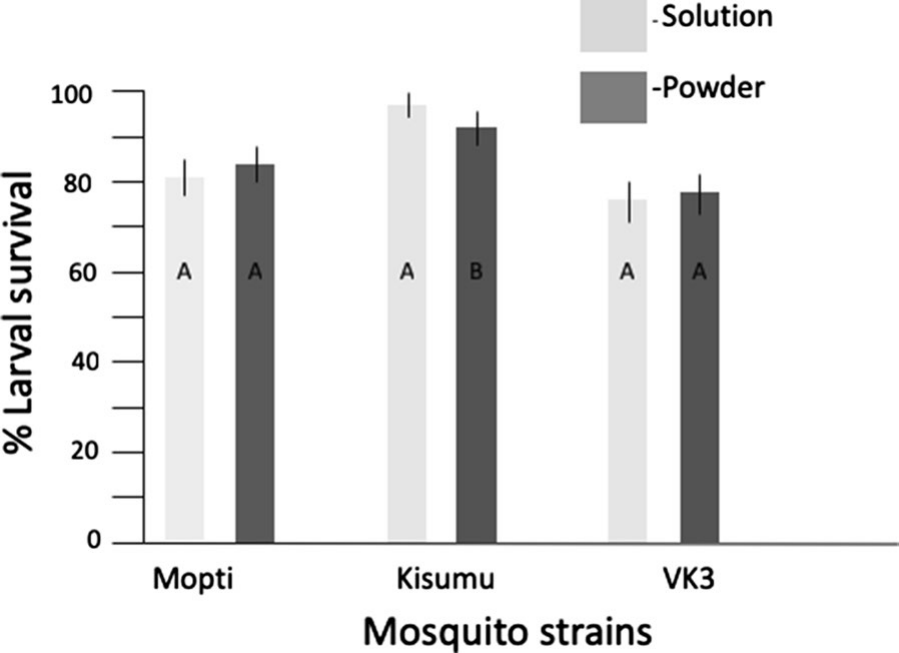
Effect of feed regimes on larval survival. The percentage larval survival for solution feed (light grey) and powder feed (dark grey) is shown for three mosquito strains (Mopti, Kisumu, VK3). Whiskers represent 95% confidence intervals. Within strains, bar plots sharing a letter are not significantly different, those with different letters are significantly different

Across all experiments *An. gambiae* larvae survived significantly (93%) better than *An. coluzzii* (Mopti: 82%; VK3: 77%) (Tables 2, 3). A full logistic regression model showed that water source had a small but significant positive effect on larval survival across all strains (*P* = 0.0405) but that its impact differed between strains (*P* = 0.0117) (Table 4). The same analyses performed within strains showed that water source improved larval survival for Kisumu and VK3 strains but not Mopti (Fig. 1, Additional file 1: Table S1). *Post-hoc* pairwise comparisons (Odds-ratio tests) revealed that mineral water significantly improved larval survival compared to deionised water (*P* = 0.0186), other water type comparisons were non-significant (Additional file 2: Table S2). Within the Kisumu strain there was 98% larval survival in mineral water compared to 93% for both mix and deionised water. VK3 strain conversely had the highest larval survival in mix water (83%) followed by deionised water (76%) with mineral water having the lowest larval survival of 72% (Fig. 1). Although overall feed type was not significant for larval survival (Table 4), there was a significant interaction between feed and strain (Table 4). For Kisumu, solution feed type resulted in significantly higher larval survival for Kisumu strain (Fig. 2, Additional file 1: Table S1).

**Table 2:** Effect of water types and feed regime on life history stages

Strain	Water type	Feed regime	% Larval survival	% Pupal survival	% Pupal mortality	% Adult emergence
Mopti (*An. coluzzii*)	Total	Total	82 (79–85) *600*		4 (3–7) *600*	78 (74–81) *600*
	Deionised	Solution	77 (68–84) *100*	96 (89–99) *77*	3 (1–8) *100*	74 (65–82) *100*
		Powder	79 (70–86) *100*	97 (91–99) *79*	2 (1–7) *100*	77 (68–84) *100*
	Mixed	Solution	82 (73–88) *100*	93 (85–97) *82*	6 (3–12) *100*	76 (67–83) *100*
		Powder	85 (77–91) *100*	94 (87–97) *85*	5 (2–11) *100*	80 (71–87) *100*
	Mineral	Solution	84 (76–90) *100*	90 (82–95) *84*	8 (4–15) *100*	76 (67–83) *100*
		Powder	87 (79–92) *100*	95 (89–98) *87*	4 (2–10) *100*	83 (74–89) *100*
	Total	Total	94 (92–96) *600*		10 (8–12) *600*	84 (81–87) *600*
Kisumu (*An. gambiae*)	Deionised	Solution	96 (90–98) *100*	89 (81–93) *96*	11 (6–19) *100*	85 (77–91) *100*
		Powder	89 (81–94) *100*	85 (77–91) *89*	13 (8–21) *100*	76 (67–83) *100*
	Mixed	Solution	96 (90–98) 100	97 (91–99) *96*	3 (1–8) *100*	93 (86–97) *100*
		Powder	90 (83–94) *100*	83 (74–90) *90*	15 (9–23) *100*	75 (66–82) *100*
	Mineral	Solution	98 (93–99) *100*	93 (86–96) *98*	7 (3–14) *100*	91 (84–95) *100*
		Powder	97 (92–99) *100*	90 (82–94) *97*	10 (6–17) *100*	87 (79–92) *100*
	Total	Total	77 (73–80) *600*		3 (2–5) *600*	74 (70–77) *600*
VK3 (*An. coluzzii*)	Deionised	Solution	69 (59–77) *100*	97 (90–99) *69*	2 (1–7) *100*	67 (57–75) *100*
		Powder	82 (73–88) *100*	95 (88– 98) *82*	4 (2–10) *100*	78 (69–85) *100*
	Mixed	Solution	87 (79–92) *100*	98 (92–99) *87*	2 (1–7) *100*	85 (77–91) *100*
		Powder	79 (70–86) *100*	95 (88–98) *79*	4 (2–10) *100*	74 (64–82) *100*
	Mineral	Solution	72 (63–80) *100*	96 (88–99) *72*	3 (1–8) *100*	69 (59–77) *100*
		Powder	72 (63–80) *100*	94 (87–98) *72*	4 (2–10) *100*	68 (58–76) *100*

*Notes*: Ninety-five percent confidence intervals are in parentheses and sample sizes are italicized. Larval survival, pupal mortality and emergence rates are calculated out of an initial number of 100 larvae (per treatment) and 600 larvae in total, and pupal survival is calculated out of a variable number of surviving larvae at pupation

**Table 3:** Effect of water types and feed on mosquito body size (wing length) and day of emergence

Strain	Water type	Feed regime	Mean wing length (mm)	Days till emergence
Mopti (*An. coluzzii*)	Deionised	Solution	3.13 (3.08–3.17) *74*	9.70 (9.54–9.87) *74*
		Powder	3.07 (3.03–3.11) *77*	9.91 (9.70–10.11) 77
	Mixed	Solution	3.19 (3.14–3.25) *76*	9.72 (9.54–9.90) *76*
		Powder	3.13 (3.09–3.18) *80*	9.68 (9.49–9.86) *80*
	Mineral	Solution	3.16 (3.11–3.22) *76*	9.50 (9.32–9.68) *76*
		Powder	3.17 (3.13–3.21) *83*	9.48 (9.34–9.63) *83*
Kisumu (*An. gambiae*)	Deionised	Solution	3.13 (3.08–3.17) *85*	10.52 (10.39–10.65) *85*
		Powder	3.18 (3.14–3.23) *76*	10.45 (10.29–10.60) *76*
	Mixed	Solution	3.23 (3.19–3.28) *93*	10.26 (10.13–10.38) *93*
		Powder	3.26 (3.21–3.29) *75*	10.01 (9.90–10.12) *75*
	Mineral	Solution	3.24 (3.18–3.27) *91*	10.32 (10.16–10.48) *91*
		Powder	3.28 (3.23–3.32) *87*	9.92 (9.79 – 10.05) *87*
VK3 (*An. coluzzii*)	Deionised	Solution	3.21 (3.16–3.27) *67*	9.60 (9.42– 9.78) *67*
		Powder	3.25 (3.21–3.28) *78*	9.69 (9.52 – 9.87) *78*
	Mixed	Solution	3.27 (3.23–3.31) *85*	9.58 (9.44– 9.74) *85*
		Powder	3.34 (3.28–3.40) *75*	9.40 (9.22– 9.58) *75*
	Mineral	Solution	3.28 (3.23–3.32) *69*	9.59 (9.41– 9.78) *69*
		Powder	3.35 (3.30–3.40) *68*	9.22 (9.03– 9.41) *68*

*Notes*: Ninety-five percent confidence intervals are in parentheses and the samples sizes, the number of surviving individuals out of an initial number of 100 larvae are italicized

**Table 4:** Logistic regressions of the overall effect of water types and feed regime on development

Parameter	Source	*df*	Likelihood ratio	*P*-value
Larval survival	Strain	2	86.74	< 0.0001***
	Water type	2	6.41	0.0405*
	Feed	1	1.88	0.1708^ns^
	Feed *vs* Strain	2	7.32	0.0258*
	Water type *vs* Strain	4	12.92	0.0117*
Pupal mortality	Strain	2	23.09	< 0.0001***
	Water type	2	0.02	0.9898^ns^
	Feed	1	1.22	0.2690^ns^
	Feed *vs* Strain	2	5.62	0.0601 ^ns^
Adult emergence	Strain	2	25.61	< 0.0001***
	Water type	2	4.11	0.1283^ns^
	Feed	1	2.51	0.1129^ns^
	Feed *vs* Strain	2	13.36	0.0013*
	Water type *vs* Strain	2	9.75	0.0448*

***P < 0.0001, **P < 0.001, P* < 0.05^ns^ > 0.05

*Abbreviation*: df, degrees of freedom

### Pupal mortality in relation to water types and feed regimes

Pupal mortality was significantly different between strains (*P* < 0.0001), with 10% mortality in Kisumu strain, 4% and 3% in Mopti and VK3 respectively (Tables 2, 4). *Post-hoc* comparisons revealed significant difference in pupal mortality between *An. gambiae* and both strains of *An. coluzzii* (Odds ratio tests: *P* < 0.0014 in both cases) (Additional file 1: Table S1). There were no significant effects of water source or feed regimes on pupal mortality (Table 4).

### Effect of water types and feed regimes on adult emergence

**Fig. 3:**
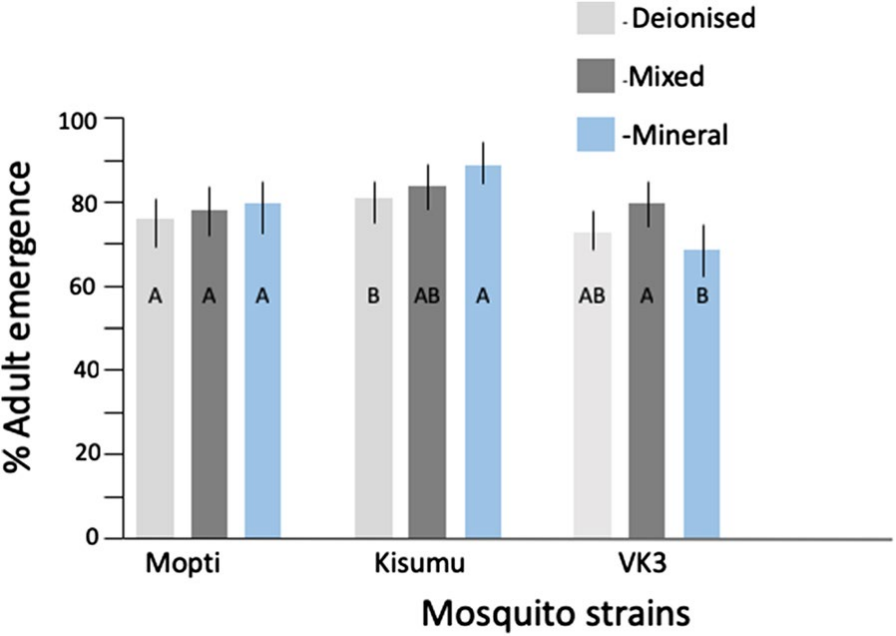
Effect of water source on adult emergence. The percentage adult emergence across three water types, deionised (light grey), mixed (dark grey) and mineral (blue) for mosquito strains, Mopti, Kisumu and VK3). Whiskers represent 95% confidence intervals. Within strains, bar plots sharing a letter are not significantly different, those with different letters are significantly different

**Fig. 4:**
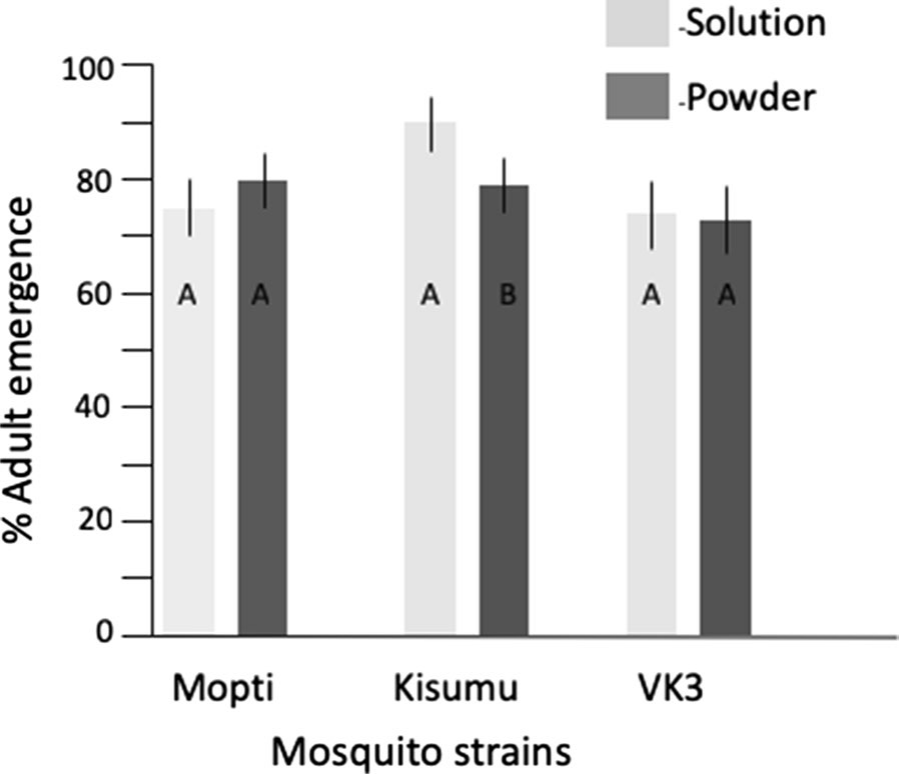
Effect of feed regimes on adult emergence. The percentage adult emergence for solution (light grey), and powder (dark grey) feed regimes across three mosquito strains (Mopti, Kisumu and VK3). Whiskers represent 95% confidence intervals. Within strains, bar plots sharing a letter are not significantly different, those with different letters are significantly different

Overall, adult emergence significantly differed among strains (*P* < 0.0001) and was also affected through the interaction of strain with feed type and water type (*P* < 0.05 in both cases) (Table 4). *Post-hoc* analysis showed that the Kisumu strain of *An. gambiae* had significantly higher adult emergence (84%) than Mopti (78%) and VK3 (74%) (Tables 2, 4, Figs. 3, 4). Within strains, water type significantly impacted adult emergence in the VK3 strain with mineral water having the lowest emergence (69%), followed by deionised water (73%), and mix (80%) (Fig. 3) but there were no effects on Mopti and Kisumu (Additional file 2: Table S2). In Kisumu, solution feed yielded 10% (*P* < 0.001) more adults compared to powder feed (Tables 2, 4, Fig. 4).

### Wing length of emerged adults in relation to water types and feed types

**Fig. 5:**
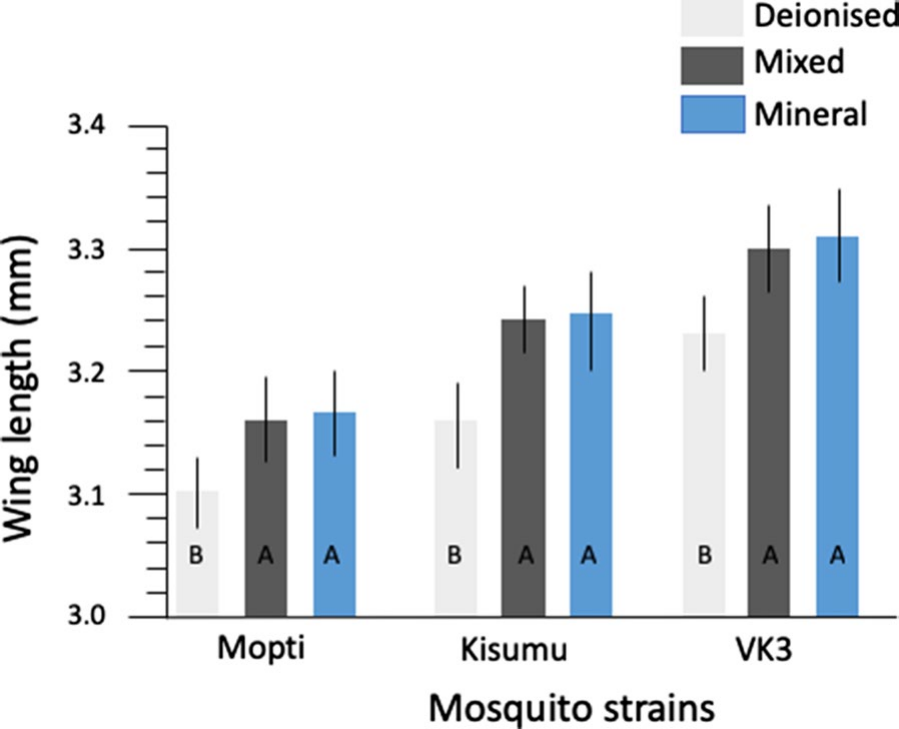
Effect of water source on wing-length. The mean wing-length for deionised (light grey), mixed (dark grey) and mineral water (blue) types for Mopti, Kisumu and VK3. Whiskers represent 95% confidence intervals. Within strains, bar plots sharing a letter are not significantly different, those with different letters are significantly different

**Fig. 6:**
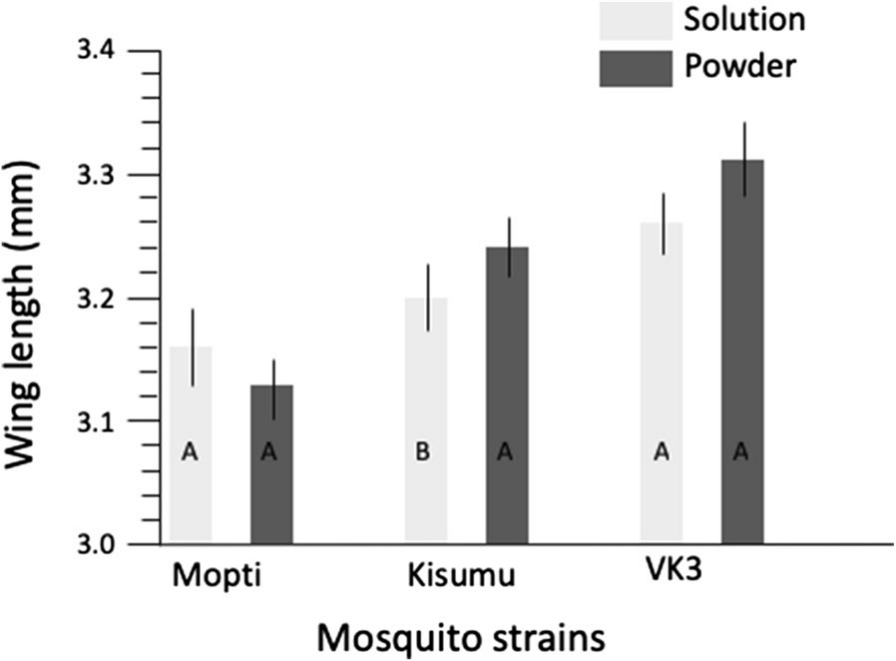
Effect of feed regimes on wing-length. The mean wing-length for solution (light grey), and powder feed (dark grey) across three mosquito strains, Mopti, Kisumu and VK3. Whiskers represent 95% confidence intervals. Within strains, bar plots sharing a letter are not significantly different, those with different letters are significantly different

Water source and feed regime significantly (*P* < 0.0001) impacted wing length of both sexes of adult mosquitoes of all strains. There were significant interactions between feed *vs* strain and sex *vs* water type (Table 5). Generally, females had longer wing-length than males. However, females from deionised water were significantly (*P* < 0.0001) smaller than those from mineral and mix water (Table 5). Similarly, males from deionised water had significantly shorter wing length compared to those from mineral water (Table 5). *Post-hoc* pairwise comparisons (Tukeyʼs tests) revealed mineral water yielded the largest adults, followed by mix, with deionised water producing the smallest mosquitoes (Additional file 3: Table S3, Fig. 5). Adults that emerged from powder feed were significantly larger than those from solution feed (Fig. 6, Table 5). Amongst strains, VK3 adults were significantly (*P* < 0.0001) the largest, then Kisumu, lastly Mopti (Fig. 6, Additional file 3: Table S3). Powder feed impacted positively on adult size for VK3 and Kisumu strain but was not significant for Mopti (Fig. 6, Additional file 3: Table S3).

**Table 5:** General linear model of the effect of water types and feed regime on wing length

Parameter	Source	*df*	*F*-ratio	*P*-value
Wing length	Strain	2	60.08	< 0.0001***
	Water type	2	26.07	< 0.0001***
	Feed	1	7.17	0.0075*
	Sex	1	146.50	< 0.0001***
	Feed *vs* Strain	2	8.60	0.0002**
	Sex *vs* Water type	2	4.23	0.0147*

***P < 0.0001, **P < 0.001, P* < 0.05^ns^ > 0.05

*Abbreviation*: df, degrees of freedom

### Development time in different water types and feed regimes

**Fig. 7:**
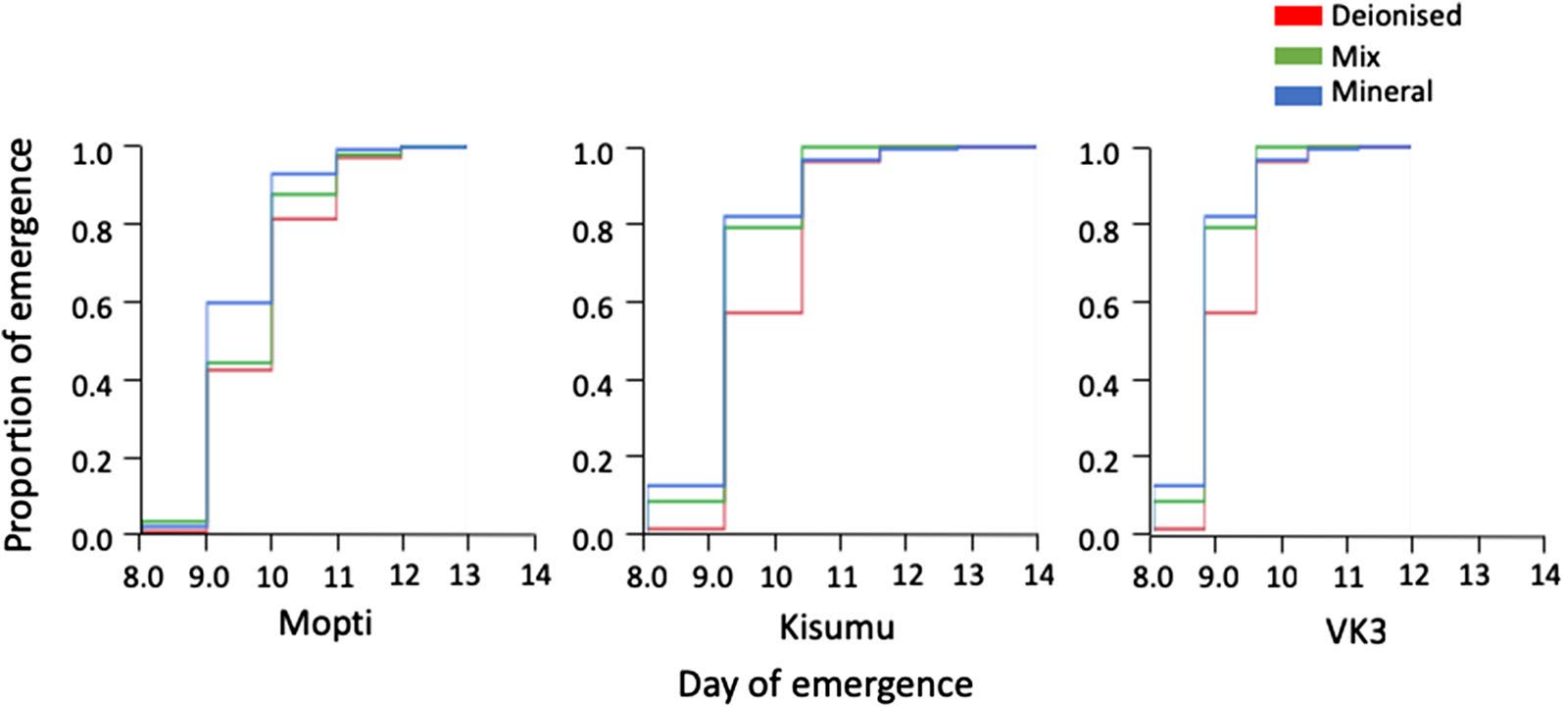
Effect of water source on development time. The mean development time for deionised (red), mixed (green) and mineral (blue) water types is shown for three mosquito strains, Mopti, Kisumu and VK3

**Fig. 8:**
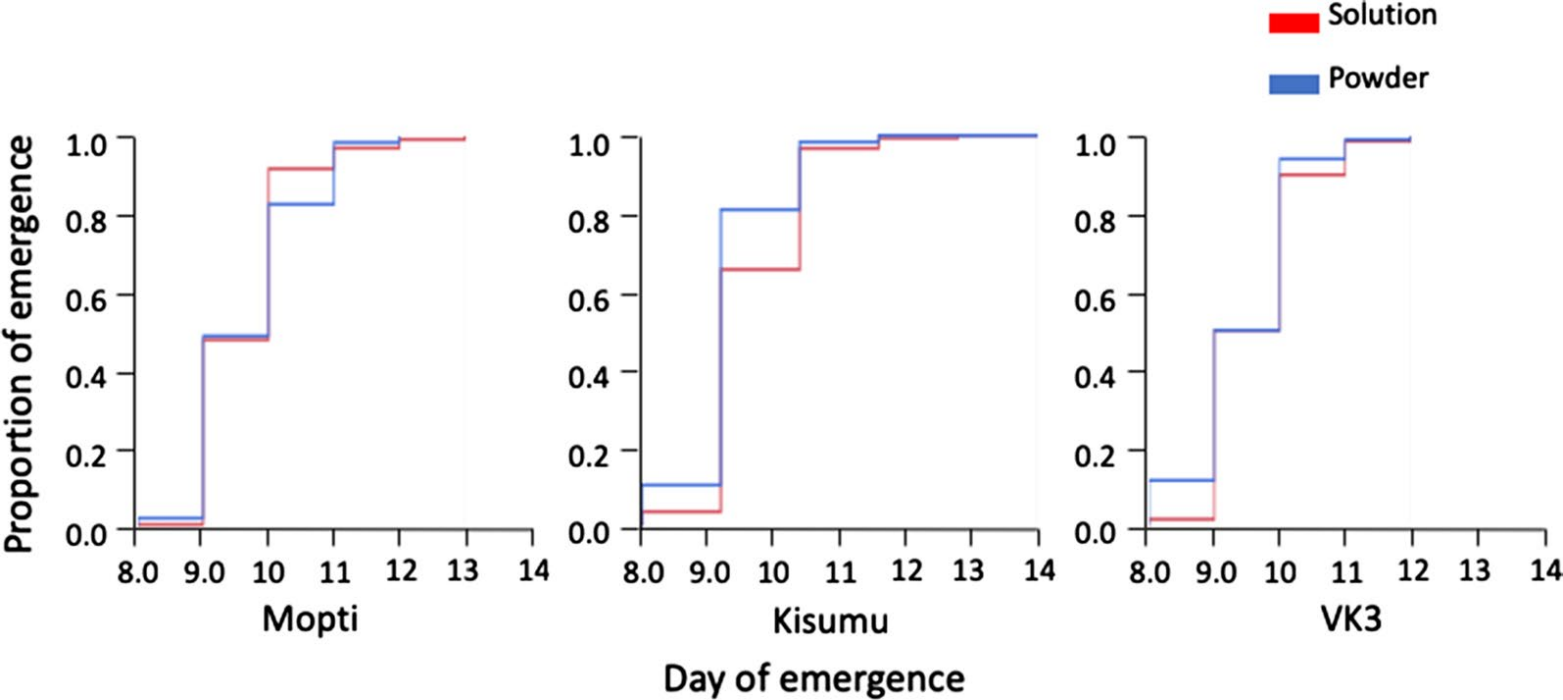
Effect of feed regimes on development time. The mean development time for solution (red) and powder (blue) feed is shown for Mopti, Kisumu and VK3

Overall, the duration of development from first instar larvae to adults was significantly impacted for all strains by water source (Cox Proportional Hazard: *P* < 0.0001) (Table 6). Mosquito development was significantly longer in deionised water compared to mix and mineral water (*P* < 0.05) (Additional file 4: Table S4, Fig. 7). Development time was not significantly different between mix and mineral water (Additional file 4: Table S4, Fig. 7). Across all water types, development time was significantly (*P* < 0.0001) longer in Kisumu strain compared to Mopti and VK3 (Table 6, Fig. 7). Feed regime did not significantly impact development time (Fig. 8).

**Table 6:** Cox Proportional-Hazard analyses on development time

Parameter	Source	*df*	Likelihood ratio	*P*-value
Day of emergence	Strain	2	79.61	< 0.0001***
	Water type	2	15.26	0.0005**
	Feed	1	2.25	0.1337^ns^

***P < 0.0001, **P < 0.001, ^ns^ > 0.05

*Abbreviation*: df, degrees of freedom

## Discussion

The results from this study show that mineral water and mixed water produced significantly larger mosquitoes compared to deionised water in the laboratory. This was true for all three strains, from the very old Kisumu strain to the recently-colonized VK3 colony. In mosquitoes, environmental conditions at the larval stage determine the size of the images [31] and larger body size is usually synonymous with higher phenotypic quality [31]. In *An. gambiae* (*s.l*.), optimal larval nutrition has been linked to female body size, fecundity and increased vectorial capacity [32]. In males, body size has been directly linked to successful mating and longevity in the wild and in the laboratory [12]. Standardization of mosquito adult size and phenotypic quality during rearing is therefore essential to ensure the reproducibility and efficiency of vector control programmes relying on mosquito releases [33]. Therefore, the results of this study suggest that the use of mineral water in mosquito culture laboratories can play an important part in achieving adequate standards of mosquito phenotypic quality. This is relevant to small-scale rearing in mosquito insectaries and may prove crucial for mass rearing protocols for larger mosquito release control programmes.

Quality and delivery of larval diet has been shown to impact on mosquito size and development [34]. In this study, the powder diet resulted in improved mosquito size, hence phenotypic quality, in two of the three strains studied, the Kisumu and VK3 strains. No significant effect of larval diet in adult size was observed for the Mopti strain. Both the Mopti and VK3 strains are of the *An. coluzzii* sibling species which is thought to be more prone to bottom feeding to avoid predators [21, 22]. Therefore, the contrasted responses to liquid feed of these two strains remains to be explained. Kisumu strain is a strain of *An. gambiae*, which is thought to prefer surface filter feeding [21]. In addition, it is a far older strain which may have become better adapted to powder food because it is widely used in insectaries.

The duration of the developmental cycle is another important parameter for mosquito rearing that is dependent on an optimal combination of feed quantity and quality, water type and ambient conditions [30]. An ideal culture timeline should be the shortest possible time required to produce good sized, long-lasting and viable mosquitoes that are able to compete with those in the wild [30]. Mineral water led to faster development, longer wing length, therefore, it stands out as the best option for improved mosquito culture. Deionised water on the other hand, had the longest developmental time and resulted in smaller mosquitoes. Small size females have been reported to have higher mortality especially after a blood feed or do not produce viable eggs [30].

Although mineral water positively impacted larval survival for Kisumu strain and had no effect on the Mopti strain, it seemed to negatively impact larval survival in the newly colonised VK3 strain. This effect might be a due to the fact that this strain is not yet adapted to the confinement conditions in the insectary or other factors. Of more importance, wing-length and development time was positively impacted by mineral water even for VK3 strain.

Mixed water and mineral water had similar impact on larval survival, adult emergence, wing length and development time despite the former having 50% less mineral content. Given that mineral water represents a significant cost, using mixed water is a cost-effective and sustainable option for mosquito rearing, especially in areas of water scarcity and with limited financial resources.

*Ad-hoc* tests revealed strong differences between the three species in how they responded to water source and feed type. Overall, *An. gambiae* (Kisumu) larvae survived significantly better than both *An. coluzzii* strains (Mopti and VK3). This higher larval survival of the *An. gambiae* Kisumu strain may be the result of 39 years of selection for insectary rearing compared to the Mopti strain of intermediate age and the young VK3 strain. The contrasted age of colonization of the strains, whilst relevant for understanding adaptations to the laboratory, limited our ability to distinguish species-specific differences such as hardness tolerance and generally prevented inferences with regards to processes that associated to larval ecological speciation between the sibling species [5, 16–19, 22].

## Conclusions

Based on the results of this study, the use of mineralized or mixed water resulting in hardness (TDS 70.5–112.2 mg/l, salinity 47.5–75.8 ppm, conductivity 100.6–160.4 μS) is recommended for rearing *An. coluzzii* and *An. gambiae* to ensure optimal qualitative yield. Powder feed is also recommended. Although the cost of mineral water may be an economic challenge in some settings, the results obtained show that a mix of mineral and deionised water produces a similar result as mineral water. Further research is needed to investigate if these gains are directly linked to mosquito longevity and fecundity as well as male competitiveness.

## Additional files

**Additional file 1: Table S1.** Odds ratios for pairwise group comparisons of the effect of water types and feed on life-cycle stages.

**Additional file 2: Table S2.** Logistic regressions of the effect of water types and feed regime on life stages within strains.

**Additional file 3: Table S3.** *Post-hoc* following General linear model, Turkey’s pairwise differences on wing length.

**Additional file 4: Table S4.** *Post-hoc* analysis following proportional-hazards fit for development time.

## References

[1] WHO (2017). World malaria report, 2017.

[2] Lees RS, Gilles JRL, Hendrichs J, Vreysen MJB, Bourtzis K (2015). Back to the future: the sterile insect technique against mosquito disease vectors. Curr Opin Insect Sci..

[3] Della Torre A, Costantini C, Besansky NJ, Caccone A, Petrarca V, Powell JR (2002). Speciation within *Anopheles gambiae*—the glass is half full. Science..

[4] Aboagye-Antwi F, Alhafez N, Weedall GD, Brothwood J, Kandola S, Paton D (2015). Experimental swap of *Anopheles gambiae*’s assortative mating preferences demonstrates key role of X-chromosome divergence island in incipient sympatric speciation. PLoS Genet.

[5] Della Torre A, Tu Z, Petrarca V (2005). On the distribution and genetic differentiation of *Anopheles gambiae s.s.* molecular forms. Insect Biochem Mol Biol.

[6] Tripet F, Touré YT, Taylor CE, Norris DE, Dolo G, Lanzaro GC (2001). DNA analysis of transferred sperm reveals significant levels of gene flow between molecular forms of *Anopheles gambiae*. Mol Ecol.

[7] Diabaté A, Dabire RK, Kengne P, Brengues C, Baldet T, Ouari A (2006). Mixed swarms of the molecular M and S forms of *Anopheles gambiae* (Diptera: Culicidae) in sympatric area from Burkina Faso. J Med Entomol.

[8] Caputo B, Santolamazza F, Vicente JL, Nwakanma DC, Jawara M, Palsson K (2011). The ‘far-west’ of *Anopheles gambiae* molecular forms. PLoS ONE.

[9] Lee Y, Marsden CD, Norris LC, Collier TC, Main BJ, Fofana A (2013). Spatiotemporal dynamics of gene flow and hybrid fitness between the M and S forms of the malaria mosquito, *Anopheles gambiae*. Proc Natl Acad Sci USA.

[10] Norris LC, Main BJ, Lee Y, Collier TC, Fofana A, Cornel AJ (2015). Adaptive introgression in an African malaria mosquito coincident with the increased usage of insecticide-treated bed nets. Proc Natl Acad Sci USA.

[11] Burt A (2014). Heritable strategies for controlling insect vectors of disease. Philos Trans R Soc Lond B Biol Sci..

[12] Diabate A, Tripet F (2015). Targeting male mosquito mating behaviour for malaria control. Parasites Vectors.

[13] Klassen W (2009). Introduction: development of the sterile insect technique for African malaria vectors. Malar J.

[14] Subra R (1981). Biology and control of *Culex pipiens quinquefasciatus* Say, 1823 (Diptera, Culicidae) with special reference to Africa. Int J Trop Insect Sci.

[15] Tene Fossog B, Antonio-Nkondjio C, Kengne P, Njiokou F, Besansky NJ, Costantini C (2013). Physiological correlates of ecological divergence along an urbanization gradient: differential tolerance to ammonia among molecular forms of the malaria mosquito *Anopheles gambiae*. BMC Ecol.

[16] Kamdem C, Tene Fossog B, Simard F, Etouna J, Ndo C, Kengne P (2012). Anthropogenic habitat disturbance and ecological divergence between incipient species of the malaria mosquito *Anopheles gambiae*. PLoS ONE.

[17] Diabaté A, Dao A, Yaro AS, Adamou A, Gonzalez R, Manoukis NC (2009). Spatial swarm segregation and reproductive isolation between the molecular forms of *Anopheles gambiae*. Proc R Soc Lond B Biol Sci..

[18] Edillo FE, Tripét F, Touré YT, Lanzaro GC, Dolo G, Taylor CE (2006). Water quality and immatures of the M and S forms of *Anopheles gambiae s.s.* and *An. arabiensis* in a Malian village. Malar J..

[19] Lehmann T, Diabate A (2008). The molecular forms of *Anopheles gambiae*: a phenotypic perspective. Infect Genet Evol..

[20] Coetzee M, Hunt RH, Wilkerson R, Della Torre A, Coulibaly MB, Besansky NJ (2013). *Anopheles coluzzii* and *Anopheles amharicus*, new members of the *Anopheles gambiae* complex. Zootaxa..

[21] Gimonneau G, Bouyer J, Morand S, Besansky NJ, Diabate A, Simard F (2010). A behavioral mechanism underlying ecological divergence in the malaria mosquito *Anopheles gambiae*. Behav Ecol..

[22] Mattah PAD, Futagbi G, Amekudzi LK, Mattah MM, De Souza DK, Kartey-Attipoe WD (2017). Diversity in breeding sites and distribution of *Anopheles* mosquitoes in selected urban areas of southern Ghana. Parasites Vectors..

[23] Yahouédo GA, Djogbénou L, Saïzonou J, Assogba BS, Makoutodé M, Gilles JRL (2014). Effect of three larval diets on larval development and male sexual performance of *Anopheles gambiae s.s*. Acta Trop..

[24] Faeza N, Webb CE, Russell RC (2012). The importance of males: larval diet and adult sugar feeding influences reproduction in *Culex molestus*. J Am Mosq Control Assoc..

[25] Takken W, Klowden MJ, Chambers GM (1998). Effect of body size on host seeking and blood meal utilization in *Anopheles gambiae sensu stricto* (Diptera: Culicidae): the disadvantage of being small. J Med Entomol..

[26] Baeshen R, Ekechukwu NE, Toure M, Paton D, Coulibaly M, Traoré SF (2014). Differential effects of inbreeding and selection on male reproductive phenotype associated with the colonization and laboratory maintenance of *Anopheles gambiae*. Malar J..

[27] Ekechukwu NE, Baeshen R, Traorè SF, Coulibaly M, Diabate A, Catteruccia F (2015). Heterosis increases fertility, fecundity, and survival of laboratory-produced F_1_ hybrid males of the malaria mosquito *Anopheles coluzzii*. G3 (Bethesda).

[28] Rubenowitz-Lundin Eva, Hiscock Kevin M. (2012). Water Hardness and Health Effects. Essentials of Medical Geology.

[29] Koella JC, Lyimo EO (1996). Variability in the relationship between weight and wing length of *Anopheles gambiae*. J Med Entomol..

[30] Araújo MS, Gil LHS (2012). Larval food quantity affects development time, survival and adult biological traits that influence the vectorial capacity of *Anopheles darlingi* under laboratory conditions. Malar J.

[31] Aboagye-Antwi F, Tripet F (2010). Effects of larval growth condition and water availability on desiccation resistance and its physiological basis in adult *Anopheles gambiae sensu stricto*. Malar J..

[32] Takken W, Smallegange RC, Vigneau AJ, Johnston V, Brown M, Mordue-Luntz AJ (2013). Larval nutrition differentially affects adult fitness and *Plasmodium* development in the malaria vectors *Anopheles gambiae* and *Anopheles stephensi*. Parasites Vectors.

[33] Valerio L, Matilda Collins C, Lees RS, Benedict MQ (2016). Benchmarking vector arthropod culture: an example using the African malaria mosquito, *Anopheles gambiae* (Diptera: Culicidae). Malar J..

[34] Linenberg I, Chri GK, Gendrin M (2016). Larval diet affects mosquito development and permissiveness to *Plasmodium* infection. Sci Rep..

