# Supplementary material for: Effect of water source and feed regime on development and phenotypic quality in Anopheles gambiae (s.l.): prospects for improved mass-rearing techniques towards release programmes
Source: Parasit Vectors. 2019 May 6;12:210. doi: 10.1186/s13071-019-3465-0 (PMC6503376; doi:10.1186/s13071-019-3465-0)
Supplement: Supplementary file 1 — Additional file 1: Table S1. Odds ratios for pairwise group comparisons of the effect of water types and feed on life-cycle stages. [file 13071_2019_3465_MOESM1_ESM.docx]

**Additional file 1: Table S1: Odds ratios for pairwise group comparisons of the effect of water types and feed on life cycle stages**

| **Parameter** | **Source** | **Level** | **Odds ratio** | **P-value** |
| --- | --- | --- | --- | --- |
| Larval survival | Strain | Kisumu vs Mopti | 0.23 | <0.0001*** |
|  |  | VK3 vs Mopti | 1.40 | 0.0214* |
|  |  | VK3 vs Kisumu | 6.03 | <0.0001*** |
|  | Water type | Mix vs Deionized | 0.74 | 0.0912^ns^ |
|  |  | Mineral vs Deionized | 0.61 | 0.0186* |
|  |  | Mineral vs Mix | 0.82 | 0.3572 ^ns^ |
|  | Feed | Powder vs Solution | 1.24 | 0.1768 ^ns^ |
|  |  |  |  |  |
| Pupal mortality | Strain | Kisumu vs Mopti | 0.46 | 0.0014* |
|  |  | VK3 vs Mopti | 1.52 | 0.1829 ^ns^ |
|  |  | VK3 vs Kisumu | 2.69 | <0.0001*** |
|  | Water type | Mix vs Deionized | 1.00 | 1.0000 ^ns^ |
|  |  | Mineral vs Deionized | 0.97 | 0.9016 ^ns^ |
|  |  | Mineral vs Mix | 0.97 | 0.9016 ^ns^ |
|  | Feed | Powder vs Solution | 0.77 | 0.2708 ^ns^ |
|  |  |  |  |  |
| Adult emergence | Strain | Kisumu vs Mopti | 1.69 | 0.0008** |
|  |  | VK3 vs Mopti | 0.80 | 0.1058 ^ns^ |
|  |  | VK3 vs Kisumu | 0.47 | <0.0001*** |
|  | Water type | Mix vs Deionized | 1.29 | 0.0707 ^ns^ |
|  |  | Mineral vs Deionized | 1.27 | 0.0971 ^ns^ |
|  |  | Mineral vs Mix | 0.98 | 0.9153 ^ns^ |
|  | Feed | Powder vs Solution | 0.83 | 0.1141 ^ns^ |

P- value: *** < 0.0001, ** < 0.001, * < 0.05, ^ns^ > 0.05
