# Supplementary material for: Effect of water source and feed regime on development and phenotypic quality in Anopheles gambiae (s.l.): prospects for improved mass-rearing techniques towards release programmes
Source: Parasit Vectors. 2019 May 6;12:210. doi: 10.1186/s13071-019-3465-0 (PMC6503376; doi:10.1186/s13071-019-3465-0)
Supplement: Supplementary file 2 — Additional file 2: Table S2. Logistic regressions of the effect of water types and feed regime on life stages within strains. [file 13071_2019_3465_MOESM2_ESM.docx]

**Additional file 2**

**Table S2: Logistic regressions of the effect of water types and feed regime on life stages within strains**

| **Parameter** | **Strain** | **Source** | **DF** | **Likelihood ratio** | **P-value** |
| --- | --- | --- | --- | --- | --- |
| Larval survival | Mopti | Water type | 2 | 4.08 | 0.1300^ns^ |
|  |  | Feed | 1 | 0.74 | 0.3900 ^ns^ |
|  | Kisumu | Water type | 2 | 6.52 | 0.0384* |
|  |  | Feed | 1 | 6.34 | 0.0118* |
|  | VK3 | Water type | 2 | 7.61 | 0.0222* |
|  |  | Feed | 1 | 0.05 | 0.8159 ^ns^ |
|  |  | Feed vs Water type | 2 | 6.66 | 0.0358* |
|  |  |  |  |  |  |
|  |  |  |  |  |  |
| Adult emergence | Mopti | Water type | 2 | 0.94 | 0.6248 ^ns^ |
|  |  | Feed | 1 | 1.89 | 0.1693 ^ns^ |
|  | Kisumu | Water type | 2 | 5.83 | 0.0543 ^ns^ |
|  |  | Feed | 1 | 12.55 | 0.0004** |
|  | VK3 | Water type | 2 | 7.04 | 0.0296* |
|  |  | Feed | 1 | 0.10 | 0.7560 ^ns^ |
|  |  | Feed vs Water type | 2 | 6.81 | 0.0331* |

P- value: *** < 0.0001, ** < 0.001, * < 0.05, ^ns^ > 0.05
