# Supplementary material for: Effect of water source and feed regime on development and phenotypic quality in Anopheles gambiae (s.l.): prospects for improved mass-rearing techniques towards release programmes
Source: Parasit Vectors. 2019 May 6;12:210. doi: 10.1186/s13071-019-3465-0 (PMC6503376; doi:10.1186/s13071-019-3465-0)
Supplement: Supplementary file 3 — Additional file 3: Table S3. Post-hoc following General linear model, Turkey’s pairwise differences on wing length. [file 13071_2019_3465_MOESM3_ESM.docx]

**Additional file 3**

**Table S3: *Post-hoc* following General linear model ,Turkey’s pairwise differences on wing length**

| **Source** | **Level** | **t-ratio** | **P-value** |
| --- | --- | --- | --- |
| Strain | Mopti vs Kisumu | -6.66 | <0.0001*** |
|  | Mopti vs VK3 | -10.88 | <0.0001*** |
|  | Kisumu vs VK3 | -4.51 | <0.0001*** |
| Water type | Deionized vs Mix | -6.44 | <0.0001*** |
|  | Deionized vs Mineral | -6.41 | <0.0001*** |
|  | Mix vs Mineral | -0.36 | 0.9294^ns^ |
| Sex * Water type | Female-Deionized vs Female-Mix | -6.21 | <0.0001*** |
|  | Female-Deionized vs Female-Mineral | -5.72 | <0.0001*** |
|  | Male-Deionized vs Male-Mineral | -3.32 | 0.0117* |

P- value: *** < 0.0001, ** < 0.001, * < 0.05, ^ns^ > 0.05. Letters
