# Supplementary material for: Effect of water source and feed regime on development and phenotypic quality in Anopheles gambiae (s.l.): prospects for improved mass-rearing techniques towards release programmes
Source: Parasit Vectors. 2019 May 6;12:210. doi: 10.1186/s13071-019-3465-0 (PMC6503376; doi:10.1186/s13071-019-3465-0)
Supplement: Supplementary file 4 — Additional file 4: Table S4. Post-hoc analysis following proportional-hazards fit for development time. [file 13071_2019_3465_MOESM4_ESM.docx]

**Additional file 4**

**Table S4: *Post-hoc* analysis following proportional-hazards fit for development time**

| **Source** | **Level** | **Risk ratio** | **P-value** |
| --- | --- | --- | --- |
| Strain | Kisumu vs Mopti | 0.65 | <0.0001*** |
|  | VK3 vs Mopti | 1.13 | 0.0713* |
|  | VK3 vs Kisumu | 1.73 | <0.0001*** |
| Water type | Mix vs Deionized | 1.17 | 0.0196* |
|  | Mineral vs Deionized | 1.29 | 0.0001** |
|  | Mineral vs Mix | 1.11 | 0.1128 ^ns^ |
| Feed | Powder vs Solution | 1.08 | 0.1337 ^ns^ |

P- value: *** < 0.0001, ** < 0.001, * < 0.05, ^ns^ > 0.05
